# Helminths of the Bluntnose Sixgill Shark, *Hexanchus griseus* (Bonnaterre, 1788), from the Strait of Messina (Sicily, Southern Italy)

**DOI:** 10.3390/ani13152405

**Published:** 2023-07-25

**Authors:** Giovanni De Benedetto, Fabiano Capparucci, Carmelo Iaria, Fabio Marino, Gabriella Gaglio

**Affiliations:** 1 Department of Veterinary Sciences, University of Messina, 98168 Messina, Italy; gdebenedetto@unime.it; 2 Department of Chemical, Biological, Pharmaceutical and Environmental Sciences, University of Messina, 98166 Messina, Italy; fcapparucci@unime.it (F.C.); ciaria@unime.it (C.I.); marinof@unime.it (F.M.)

**Keywords:** elasmobranch, *Nybelinia* sp., *Phyllobotrium sinuosiceps*, *Otodistomum veliporum*, central Mediterranean Sea

## Abstract

**Simple Summary:**

*Hexanchus griseus* (Bonnaterre, 1788) is a deep-water shark usually found dead along the Sicilian coasts; as few samples have been found, the parasitic fauna of *H. griseus* has not been well described so far. The helminth fauna of this species is described in this study. One *H. griseus* specimen was referred to our laboratory for necropsy and parasitological analysis. After necropsy, the specimen’s gills, skin and organs were investigated for parasites. Three species of helminths were found in one studied female specimen of *Hexanchus griseus*, namely, two cestode species (*Phyllobothrium sinuosiceps* and *Nybelinia* sp., larvae) and one trematode (*Otodistomum veliporum*). No other parasite taxa were found in the celomic organs. This study improves current knowledge of the helminth fauna of *H. griseus* from the central Mediterranean Sea.

**Abstract:**

Bluntnose sixgill shark, *Hexanchus griseus* (Bonnaterre, 1788), is a little-known elasmobranch in the Mediterranean Sea. Given the lack of information about *H. griseus*, the aim of our study was to describe the helminth fauna of this species. In March 2023, one *H. griseus* juvenile female specimen was found off the coast of Messina (Italy) and referred by the Italian Coast Guard to our laboratory for necropsy and parasitological evaluation. After necropsy, the specimen’s gills, stomach and spiral valve were investigated for parasite presence. All collected parasites were stored in 70% ethanol for routine parasitological analysis. No lesions due to parasites were found in the gills or skin. Three species of helminths were found in one studied female specimen of *Hexanchus griseus*, namely, two cestode species (*Phyllobothrium sinuosiceps* and *Nybelinia* sp., larvae) and one trematode (*Otodistomum veliporum)*. Among them, five Trypanorhyncha plerocercoid larvae were found attached to the stomach mucosa, and six adult cestodes and one digenean trematode were collected from the spiral valve. No other parasite taxa were found in the celomic organs. This study reports new information regarding the parasitic fauna of *H. griseus* from the central Mediterranean Sea.

## 1. Introduction

Mediterranean shark ecosystems are threatened by many factors [1]. Among them, pollution and climate change, as well as human contribution via non-regulated fishing, must to be considered [2]. Two species belonging to the genus *Hexanchus* (Rafinesque, 1810) (Hexanchiformes, Hexanchidae) have been described so far [3]. Among them, the bluntnose sixgill shark, *Hexanchus griseus* (Bonnaterre, 1788) has been reported in the Mediterranean Basin, representing one of the most widespread large sharks inhabiting this area [4]. The other species, *Hexanchus nakamurai* (Teng, 1962), is less frequently reported than *H. griseus* in the Mediterranean Sea [3]. The total length at birth of Mediterranean *H. griseus* is between 60 and 75 cm; the adult maximum length is between 480 and 500 cm [5,6]. Female specimens reach sexual maturity between 300 and 400 cm; male specimens mature when they reach a total length of 270 cm [7]. In the Mediterranean area, *H. griseus* is distributed in the temperate seas; adult specimens are usually found between 200 and 2500 m deep [8,9], sometimes reaching the surface. Juvenile specimens have been frequently observed along the coasts, where waters are not as deep [10]. *Hexanchus griseus* is characterized by nocturnal feeding habits [11]. The diet of this elasmobranch, according to different development stages, includes diverse species of small elasmobranch, benthic and pelagic teleosts, cephalopods, crustaceans, gastropod molluscs, as well as small marine mammals and dead seawater animals [11,12]. This elasmobranch is considered a common bycatch species worldwide, caught mainly by the usual professional fishing techniques, including bottom trawls, trammel nets, hand lines and longlines [13]. A study conducted by Nuez and colleagues [14] highlighted that in Italy (south Mediterranean Sea), the most used fishing technique is the trammel net. Moreover, the migratory habit of this species [15], characterized by continuous movement between different areas, increases the difficulty of describing *H. griseus* adaptation and behaviour. Due to a continuous reduction in *H. griseus* numbers, mainly caused by fishing pressure worldwide, this species is currently listed as “Near Threatened” on the Red List, International Union for Conservation of Nature (IUCN). Parasites play a key role in natural ecosystems, carrying out many functions of both structure and performance, and regulating host population dynamics [16,17,18]. Many parasites are directly connected with their hosts; parasitizing hosts are usually preyed upon by other organisms, considered intermediate hosts, which allow the correct development and completion of their life cycles [19,20]. Tapeworms, considered the second most representative group of flatworms [21], are obligate internal parasites, described in both higher and lower vertebrates. Their complex life cycles include one or more intermediate hosts, which include many animals (gastropods, molluscs, arthropods, fish, elasmobranchs and mammals). Intermediate stage transmission occurs exclusively through food; tapeworms become adults in organisms considered to be at the top of the food chain, including sharks [22]. Tapeworms of aquatic organisms are important, as they parasitize both elasmobranchs and teleosts, at least in an intermediate or definitive state [23,24]. As they are strictly specific with their intermediate and final hosts, tapeworms are considered excellent models of their hosts’ development, and for the evaluation of host–parasite interactions and coevolution [24]. Among them, adult tapeworm specimens identified as *Phyllobothrium dohrni* (Örley, 1885), *Crossobothrium laciniatum* (Linton, 1889), *Phyllobothrium sinuosiceps* (Williams, 1959) and *Grillotia heptanchi* (Vaullegeard, 1899) have been reported attached to the mucosa of the anterior portion of the *H. griseus* spiral valve worldwide [23,25,26,27]. Cartilaginous fish are also considered as hosts of other parasite groups, including Myxozoa [28,29], as reported by Lisnerová et al. [30], describing the presence of *Chloromyxum bulliti* n. sp. in *H. griseus* sampled along the African coasts. Other parasite taxa reported in this species include third stage nematode larvae belonging to the species *Anisakis simplex* (Anisakidae), which were found in three *H. griseus* specimens, within the stomach and the spiral valve lumen, as well as, in one case, encysted in the stomach wall [31]. Adult digenean trematodes, originally morphologically identified as *Otodistomum plicatum,* were found in the pyloric region of the stomach of one *H. griseus* specimen caught off the Washington coast (United States) [32], and subsequently renamed *Otodistomum veliporum* by Gibson and Bray [33]. Acanthocephalan belonging to the species *Corynosoma australe* and *Corynosoma* spp. were found attached to the stomach mucosa of *H. griseus* caught off the Southern coasts of Brazil [34]. One hundred and fifty copepods, morphologically identified as *Perissopus oblongus* (Wilson, 1908), were found attached to *H. griseus* skin, sampled off Cape Recife, South Africa [35]. The aim of this study was to describe the helminth parasite fauna of an *H. griseus* specimen collected along the coast of Messina, an area characterized by a particular ecosystem, where it is not common to find this type of shark.

## 2. Materials and Methods

### 2.1. Sampling and Macroscopic Evaluation

In March 2023, one *H. griseus* specimen was found by an Italian Coast Guard Unit off the coast of Messina (Italy) (38°15′52.8″ N 15°39′04.3″ E) and referred to the laboratory of the Department of Chemical, Biological, Pharmaceutical and Environmental Sciences (ChiBioFarAm), University of Messina (Italy) for necropsy and parasitological evaluation.

During necropsy, no macroscopic lesions attributable to traumatic injuries or infectious diseases were found in the celomic cavity. Moreover, the stomach and spiral valve appeared completely empty; no food residue attributable to prey was found inside the lumen. Biometric indices of body weight (BW) and total length (TL) were recorded; the *Hexanchus griseus’* measurements were 65 kg and 237 cm, respectively. Total length, body weight and gonadal development allowed identification of the specimen as a juvenile female. Skin and gill scrapings and biopsies were performed to detect the presence of flukes and protozoa; the stomach and spiral valve were previously opened using scissors, macroscopically observed for the presence of parasites, and then, a stomach and spiral valve mucosa gentle scraping was performed with the aid of a glass slide to collect all gastrointestinal contents. The material collected was first rinsed three times using saline solution, and then observed in a petri dish under a stereo microscope (SteREO Discovery, V 12 Zeiss, Jena, Germany). All collected parasites were stored in 70% ethanol for further analysis.

### 2.2. Parasitological Evaluation

Some parasite specimens were clarified in glycerine for 24 h; selected parasites were stained using Semichon’s carmine red technique as described by Cable [36], properly modified according to sample size. Briefly, the samples were stained in acetocarmine red solution for at least 12 h, bleached in a chloride acid–alcohol solution at 1%, washed in distilled water to remove the hydrochloric acid, dehydrated in increasing alcoholic solutions for 5 min each (70°, 80°, 90°, 95° and 100°), diaphanized in clove oil for 1 h, and then mounted using Canada balsam. Parasite morphological identification was carried out under an optic microscope (Axioskop 2 plus Zeiss, Jena, Germany) following the suggested keys [33,37,38]. All pictures were acquired with the aid of a digital camera (Axiocam Mrc Zeiss, Jena, Germany) and a digital acquisition system (Axiovision Zeiss, Jena, Germany).

### 2.3. Scanning Electron Microscopy Evaluation

Some specimens were evaluated using scanning electron microscopy (SEM). Briefly, four parasites were dehydrated in increasing alcoholic solutions for 20 min each (70°, 75°, 80°, 85°, 90°, 95° and 100°), dried according to the critical point method, sputtered using a palladium gold layer (20 nm ± 5%) and observed via SEM (EVO MA-10, Zeiss, Jena, Germany).

## 3. Results

Three species of helminths were found in one studied female specimen of *Hexanchus griseus*, namely, two cestode species (*Phyllobothrium sinuosiceps* and *Nybelinia* sp., larvae) and one trematode (*Otodistomum veliporum*). During external evaluation, some parasites, morphologically belonging to cestodes, were collected directly from the cloaca. During parasitological evaluation, no macroscopic lesions attributable to parasites were observed in the skin or gills. No microscopic parasites were found from the gill scrapings or biopsy. Upon opening the stomach, tapeworm larvae were macroscopically observed attached to the mucosa layer in the pyloric area, and tapeworm adults were observed in the spiral valve. Upon observation of the stomach and spiral valve contents, five Trypanorhyncha plerocercoid larvae were found in the stomach, and six adult cestodes and one adult digenean trematode were found in the spiral valve. Morphological characteristics allowed identification of the Trypanorhyncha larvae as *Nybelinia* sp. Morphological features of the scolex and the distal portion of the strobila allowed identification of all adult cestodes as *Phyllobothrium sinuosiceps*; adult digenean trematodes were morphologically identified as belonging to the genus *Otodistomum*. No other parasite taxa were found in the celomic organs.

Morphometric feature measurements of mean length (ML) and mean width (MW) ± standard deviation were taken on all specimens and reported in micrometers (µm) below.

### 3.1. Nybelinia sp., Larvae (Trypanorhyncha: Tentaculariidae)

**Figure 1:**
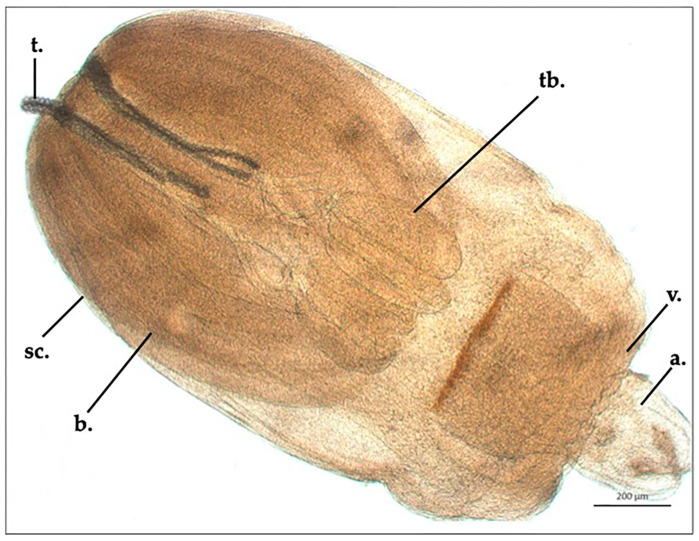
*Nybelinia* sp. (Poche, 1926) larvae collected from *Hexanchus griseus* stomach after glycerine diaphanization (t., tentacle; sc., scolex; b., bothria; tb., tentacular bulbs; v., velum; a., appendix).

*Nybelinia* sp. plerocercoid appeared pyriform in shape; its ML and MW were 2324.6 ± 23.4 µm and 1227.6 ± 17.3 µm, respectively. The scolex’s ML was 1354.5 ± 6.4 µm, and two bothria were present (ML: 1298 ± 14.7 µm, MW: 567 ± 5.8 µm). The tentacular bulb’s length was 423 ± 8.2 µm. The appendix’s ML was 8118 ± 2.8 µm, and the velum’s ML was 488.5 ± 4.9 µm. Four cylindrical tentacles, in only one specimen partially everted, were present, of which everted parts were 544 to 586 µm long, and the tentacular widths were 28.6 µm (basal area), 30.9 µm (metabasal area) and 28.4 µm (apical area). Tentacles were covered with uncinate hooks disposed in oblique ringlet lines, which were 11.3 µm long and 8.4 µm wide (basal area), 14.8 µm long and 12.5 µm wide (metabasal area), and 13.5 µm long and 10.2 µm wide (apical area) (Figure 1).

### 3.2. Phyllobothrium sinuosiceps (Phyllobothriidea: Phyllobothriidae) Adult Specimen

**Figure 2:**
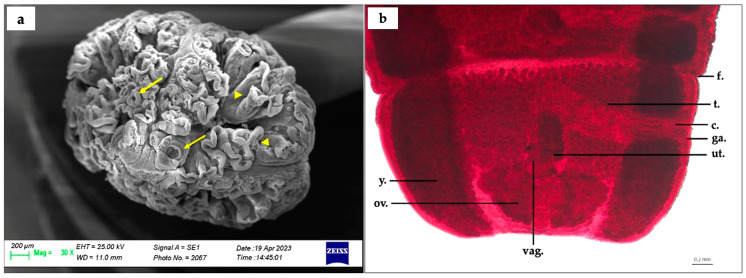
*Phyllobothrium sinuosiceps* (Williams, 1959) collected from *H. griseus* spiral valve: (**a**) *P. sinuosiceps* scolex, scanning electric microscopy observation, apical sucker (arrow); curled margin of the scolex folds (arrowhead) and (**b**) *P. sinuosiceps* terminal segment after Semichon’s carmine staining (y., yolk sack; ov., ovary; vag., vagina; ut., uterus; f., posterior flap of segment; ga., genital atrium; c., cirrus; t., testis).

The scolex appeared spherical in shape (ML: 3130.7 ± 27.5 µm, MW: 2386.5 ± 20.5 µm), and presented with a series of folds, arranged in random and different positions. It was completely or partially surrounded by two to four folds. In general, these appeared as major folds, with two or three divisions; all the margins of major and minor folds appeared curled. Caudal folds of the scolex overlapped the anterior part of the strobila. Four small suckers, located at the top of the upper portion of the scolex and of variable size (MW: 304.5 ± 0.7 µm), were present. The strobila’s MW was 2046 ± 11.3 µm, and the cuticle appeared scaly for the entire strobila length; at the posterior end, the examined mature segments varied in shape, being generally wider than long (ML: 1639.8 ± 31.4 µm, MW: 3024 ± 7.1 µm). The excretory system of the scolex appeared simple in structure; the cirrus sac’s ML and MW were 1401.5 ± 0.7 µm and 397 ± 1.4 µm, respectively. Regarding the reproductive system, the testes were not counted, but appeared spherical in shape (MW: 49.7 ± 2.5 µm) and disposed in the middle of the mature segment, between the yolk glands, and associated with the vagina and cirrus sac. The vagina was characterized by thin walls and located in the middle of the uterus, directly connected to the cirrus sac. The uterus was localized above the ovary, in the middle region of the mature segment (ML: 791 ± 7.1 µm, MW: 489 ± 1.4 µm); the ovary was leaf-like in shape (ML: 1272.3 ± 4.3 µm, MW: 474.3 ± 3.5 µm) and set in the middle posterior part of the segment, between the yolk glands. The posterior flap of the evaluated segments did not appear clearly marked (Figure 2a,b).

### 3.3. The trematode Otodistomum veliporum Creplin, 1837 Immature Specimen

**Figure 3:**
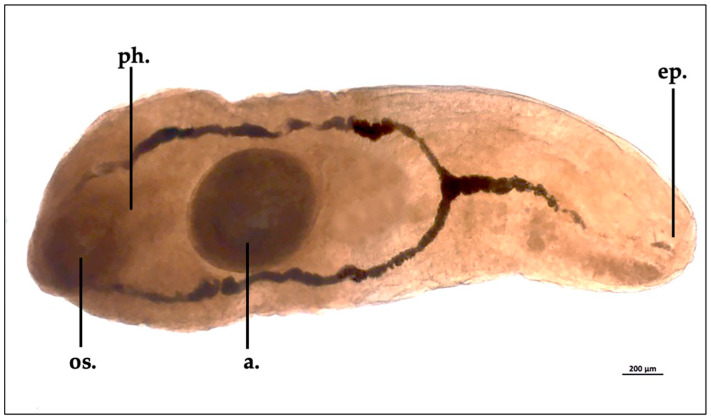
*Otodistomum veliporum* collected from *H. griseus* spiral valve (os., oral sucker; ph., pharynx; a., acetabulum; ep., excretory pore).

Morphological features of the only specimen collected were a length of 3840 µm and a width of 1360 µm; the oral sucker width was 480 µm, and the acetabulum measured 748 µm in length. The yellow-whitish external cuticle appeared non-spinous. The *Otodistomum veliporum* specimen showed the pharynx closer to the oral sucker than the acetabulum (Figure 3).

## 4. Discussion

In this study, parasitological evaluation of *H. griseus* (caught off the Sicilian coasts) helminth fauna was carried out, describing three parasites in this area. Considering the *H. griseus* development stages described by Capapé et al. [5,6], biometric indices and gonadal development, the specimen examined in this study was considered juvenile. The parasite fauna found were partially in agreement with some previous findings.

Among them, *Nybelinia* sp. larvae have been reported in several elasmobranch species from the Mediterranean Sea [39]. Regarding the epidemiological indices of this species, low prevalence was reported by Pascual et al. [40,41] in the lumen and attached to the digestive tract or in the mantle cavity of the cephalopods *Illex coindetii* (Vérany, 1839) and *Todaropsis eblanae* (Ball, 1841), which are considered intermediate hosts in the Mediterranean Sea and Atlantic Ocean; these data were confirmed by Gestal [42]. In our study, we partially confirm this data, due to the empty stomach and spiral valve of the examined *H. griseus*. The Tentacularidae life cycle generally includes elasmobranchs as a definitive host, as reported by Mulas and colleagues [43], who carried out a survey of *Hexanchus griseus* and *Scyliorhinus canicula* sampled from the Central–Western Mediterranean Sea; these data were not confirmed by our results, due to the *Nybelinia* sp. plerocercoid findings. *Nybelinia* sp. larvae have been reported by Reinero et al. [39] in Lesser Spotted Dogfish (*Scyliorhinus canicula*) from the Central Tyrrhenian Sea with a prevalence of 24%; in that study, parasites were localized mainly in the spiral valve and stomach. However, in our study, *Nybelinia* sp. larvae were found only in the *H. griseus* stomach, confirming this tract as the elective localization of these parasites. According to Palm and Walter [44], who found three adult cestodes identified as belonging to the genus *Nybelinia*, *H. griseus* can be considered as a definitive host of these parasites. Instead, according to our results, we can speculate that this elasmobranch may be considered as an intermediate host of *Nybelinia* sp. Moreover, according to Hart’s [45] Pacific Ocean study, Tetrarhynchidea metacestodes can be found in *H. griseus* specimens, attached to the spiral valve wall, just below the pyloric region. Our results suggest that this finding can be considered as an erratic localization.

*Phyllobothrium sinuosiceps*, a tapeworm characterized by a complex life cycle, is considered a parasite that usually inhabits the spiral valve of various elasmobranchs. Thanks to its highly adaptive specialization to the unique environment of the shark digestive tract, it should also be considered from an ecological point of view [46]. *Phyllobothrium sinuosiceps* (as well as other cestodes) do not typically cause direct injuries to their hosts; however, a heavy parasite load may have indirect effects on host health. Indeed, possible lesions in the shark spiral valve due to a parasite may cause acute inflammation, with a consequent reduction in nutrient absorption, which is potentially dangerous for correct shark development [47]. In our case, the low parasite load collected, according to the general growth condition of the host, can exclude parasite infection as causative of death. Moreover, according to Caira et al. [46], the presence of *P. sinuosiceps* in sharks highlights the close ecological connections within marine ecosystems. As sharks have been considered predators in their habitat, feeding habits and interactions between these animals and their prey play a key role in the transmission and adaptation of their parasites [48]. To better understand the dynamics concerning these organisms, it is necessary to evaluate the food chain and the status of the ecosystem; unfortunately, in our case, the absence of food debris in the gastrointestinal tract meant this was not feasible. Adult *Phyllobothrium* sp. specimens have been found in *Carcharhinus limbatus* (Müller and Henle, 1839) and *Carcharhinus obscurus* (Lesueur, 1818) by Pramanik and colleagues [49]; among other shark species, *Sphyrna zygaena* (Linnaeus, 1758) [50], *Galeocerdo cuvier* (Péron and Lesueur, 1822) [51] and *Mustelus mustelus* (Linnaeus, 1758) [52] sampled from different areas, including the Mediterranean Sea, were positive for *Phyllobothrium* sp. found in the stomach and spiral valve. Our results confirm that this tapeworm is strictly related to many elasmobranch species from the Mediterranean Sea, as well as other basins worldwide. In our study, six specimens of *P. sinuosiceps* were collected. Morphological identification was performed following the morphological description reported by Williams [37] in *H. griseus*. This author described *P. sinuosiceps* for the first time in *H. griseus* caught in the Celtic Sea. According to our results, we can confirm the same morphological features in *H. griseus* from different regions. Henderson [31] reported *P. sinuosiceps* in *H. griseus* from the west of Ireland, localized in the stomach and spiral valve, but also encysted in the stomach wall; as previously discussed regarding other parasites, such as *Nybelinia* sp., and considering the habit of *P. sinuosiceps* to infect the spiral valve, we can consider the stomach as an erratic localization for this parasite. *Phyllobothrium sinuosiceps* infection intensity can be variable in *H. griseus*. Studies have shown that the intensity of this tapeworm can be relatively high in some populations of *H. griseus* [53]. In our case, the number of adult cestodes found may have been underestimated due to the possible emission of parasites before the host was found.

Based on morphometric features and the oral sucker and acetabulum size, the Digenean trematode reported was identified as belonging to the genus *Otodistomum* (Stafford, 1904, Family Azygiidae), confirming *H. griseus* as a definitive host, as previously reported by Kay [32]. *Otodistomum veliporum* has been described from sub-tropical, temperate and polar regions [54,55], including the Atlantic Ocean and the Central Mediterranean Sea, where Gibson and Bray [33] and Gibson [56] reported *O. veliporum* as a parasite of many bony fish and elasmobranchs, such as *Squalus acanthias* (Linnaeus, 1758)*, Raja radiata* (Donovan, 1808)*, Centroscyllium fabricii* (Reinhardt, 1825) and *Pristiophorus cirratus* (Latham, 1794). Our results allowed inclusion of *H. griseus* among the possible definitive hosts of *O. veliporum* inhabiting the Mediterranean Sea. Among other species belonging to the genus *Otodistomum,* up to one hundred specimens have been found in *H. griseus* (caught on the Pacific Ocean) stomachs’ pyloric region [32]. According to our results, the only *O. veliporum* specimen found suggests a low density of populations of intermediate hosts in the diet of *H. griseus* present in the Strait of Messina. Recently, Sperone and Milazzo [57] described *O. veliporum* in kitefin shark (*Dalatias licha*, Bonnaterre, 1788) from the Southern Tyrrhenian Sea (Central Mediterranean Sea). In this case, the authors reported one *O. veliporum* specimen, attached to the mucosa of a *D. licha* spiracle. This unusual localization, compared to our results, could be attributable to a postmortem accidental parasite movement from the gastrointestinal tract to the spiracle. Another *Otodistomum* sp. single finding was reported by Espínola and Novelo [58] in southern lanternshark *(Etmopterus granulosus*, Günther, 1880) collected from the southeastern Pacific Ocean. Similarly, in our study, as previously discussed, the single specimen found may have be related to the low availability of intermediate hosts.

## 5. Conclusions

This study reports a new parasitological evaluation in *H. griseus* from the Central Mediterranean Sea. The difference between the Strait of Messina and other study areas, where these parasites have already been reported, lies mainly in the conformation of teleost and cephalopods varieties as intermediate hosts. Moreover, the movement of these large elasmobranchs between the Ionian and Tyrrhenian Seas, including their daily vertical migration, adds many variables to this type of survey, making it difficult to evaluate possible host–parasite interactions.

Therefore, the results reported in this study confirm the bluntnose sixgill shark as a definitive host of *P. sinuosiceps* and *O. veliporum*, and provide new data about this species as an intermediate host of *Nybelinia* sp. Finally, we highlight the need to carry out further studies of the epidemiology of these parasite species in the central Mediterranean Sea.

## Data Availability

Not applicable.
